# Venue-Based Networks May Underpin HCV Transmissions amongst HIV-Infected Gay and Bisexual Men

**DOI:** 10.1371/journal.pone.0162002

**Published:** 2016-09-01

**Authors:** Daniel Bradshaw, Jayna Raghwani, Brendan Jacka, Rachel Sacks-Davis, Francois Lamoury, Ian Down, Garrett Prestage, Tanya L. Applegate, Margaret Hellard, Joe Sasadeusz, Gregory J. Dore, Oliver G. Pybus, Gail V. Matthews, Mark Danta

**Affiliations:** 1 The Kirby Institute for Infection and Immunity in Society, UNSW Australia, Sydney, NSW, Australia; 2 Department of HIV/GU Medicine, Brighton and Sussex University Hospitals NHS Trust, Brighton, United Kingdom; 3 Department of Zoology, Oxford University, Oxford, United Kingdom; 4 Burnet Institute, Melbourne University, Melbourne, Victoria, Australia; 5 Department of Infectious Diseases, Royal Melbourne Hospital, Melbourne, Victoria, Australia; 6 St Vincent’s Clinical School, UNSW Australia, Sydney, NSW, Australia; Agencia de Salut Publica de Barcelona, SPAIN

## Abstract

**Background:**

This study aimed to investigate the potential influence of venue-based networks on HCV transmission in HIV-positive gay and bisexual men (GBM).

**Methods:**

This was a prospectively recruited cohort of HIV-infected GBM with recently-acquired HCV infection resident in Melbourne and Sydney. Clinical and demographic data were collected together with blood samples for HCV sequencing. Phylogenies were inferred and clusters of individuals infected with HCV with genetic sequence homology were identified. Venues used for sourcing sexual partners were identified; sourcing partners from the same venue was considered a potential social link. Using the Jaccard similarity coefficient, associations were identified between the network of sites where men sourced sex partners and transmission relationships as defined by phylogenetic clustering.

**Results:**

Forty individuals were recruited, of whom 62.5% were considered to have sexually- and 37.5% IDU-acquired HCV. Venue use was consistent with men being members of a more sexually adventurous gay community subculture. Six phylogenetically-determined pairs or clusters were identified, comprising fifteen (15/28, 53.6%) individuals. Participants belonging to phylogenetic clusters were observed within the same networks. There was a significant correlation between the network and phylogenetic clustering when both cities were considered simultaneously (p = 0.005), raising the possibility that social connections may be important for HCV transmissions.

**Conclusions:**

Venue-based network elicitation is a promising approach for elucidating HCV transmissions amongst HIV-infected GBM. Public health approaches targeting individuals and venues prominent within networks may reduce onward HCV transmission.

## Introduction

Many countries have reported rising numbers of HIV-positive gay and bisexual men (GBM) with acute hepatitis C virus infection (AHCV) over the past decade [1–4], with an estimated seroconversion rate of 0.53/100 person-years, as well as high rates of reinfection [5, 6].Many infected individuals deny a history of injecting drug use (IDU) and transmission is often considered to occur through the permucosal, mostly sexual, route. Factors associated with sexual transmission include both biological (coinfection with HIV and other sexually transmitted infections (STI)) and behavioural (high risk sex and permucosally-administered recreational drugs)[7–9].

Venue-based network analysis has previously shown that the interaction of individuals with social venues may be important for HCV transmission amongst people who inject drugs (PWID) and for gonorrhoea transmission within a localised heterosexual outbreak. Such an approach has enabled the identification of characteristics of individuals who play important roles within the network, for example those interacting with multiple venues, and who therefore may pose increased risk of onward transmission [10, 11]. An alternative technique for investigating transmission dynamics is phylogenetic analysis, in which the shared history of viral strains is reconstructed through analyses of genetic sequence homology.

HCV transmission is particularly prevalent amongst HIV-positive GBM practising shared high risk behaviours [7, 8, 12, 13]. It is widely known that GBM identify sexual and drug-taking partners through different virtual (mobile phone aps, websites) and physical (sex on premises venues, bars/clubs etc) venues, with the use of online venues having become fairly ubiquitous amongst GBM [14]. Venue usage is associated with membership of different gay community subcultures and likely indicates increased social and sexual contact with other men who similarly use those venues. Further, men engaged in a sexually adventurous subculture report behaviours which increases their risk of acquisition of sexually-transmitted infections (STIs) and most new HIV infections occur in this group [15, 16]. Venue-based networks involving GBM may also be considered examples of sexual fields, an arena of social life in which individuals seek sexual partners and vie for sexual status. In this model, the sexual field describes a bi-directional relationship between sexual preferences and sexual subcultures/venues, such that men will choose to participate in particular subcultures based on their sexual preferences, whilst equally, sexual preferences/desires will be influenced by the subcultures/venues in which they participate. In this way, the sexual field may play a role in making generally desirable, for example, certain high risk behaviours which increase STI transmission [17].

Venue-based network analysis identifies potential social links between individuals and therefore potential paths of HCV transmission but cannot identify actual chains of viral transmission. Conversely, phylogenetics identifies the likely pathways through which HCV has actually been transmitted between individuals, but not the social connections between them. Combining these techniques has the potential to link viral transmissions with corresponding social behaviours. A previous study has shown the importance of social networks in influencing HCV transmission amongst PWID through the identification of a correlation between phylogenetic clustering and injecting relationships [18].

Venue—based networks, as indicative of membership of particular subcultures, are likely to be important for HCV transmissions amongst GBM, but this has not previously been investigated. The current study aimed to examine HCV transmission patterns within HIV-positive GBM in Australia and to investigate the role of venue-based networks in this phenomenon.

## Methods

### Recruitment

This was a prospective cohort study of HIV-positive GBM with recently-acquired HCV. Recent HCV acquisition was determined according to previously-outlined criteria [19]. Individuals were eligible if they had tested positive for anti-HCV antibody or HCV RNA ≤24 weeks before screening and had either (1) acute clinical infection (defined as symptomatic seroconversion or an alanine aminotransferase level of >10 times the upper limit of normal, excluding other causes of acute hepatitis, within 24 weeks of the initial positive anti-HCV antibody) or (2) asymptomatic HCV infection with seroconversion (defined as a negative result of a test for anti-HCV antibody during the 48 weeks before the initial positive result of a test for anti-HCV antibody). The maximum estimated duration of infection was therefore 48 weeks (S1 File). Recruitment was through the viral hepatitis clinics of St Vincent’s Hospital, Sydney and the Alfred Hospital, Melbourne, Australia between 2008 and 2013. Recruits provided written informed consent. Participants completed questionnaires regarding clinical and demographic data, and sexual and drug taking behaviour (S2 File). Detailed interviews were also conducted in which participants were asked both open and specific, closed questions regarding venues used for sourcing sexual partners over the six months prior to the study visit (S3 File). Participants reporting IDU within the preceding month were additionally asked questions about their injecting practice. Data were also gathered from medical notes review. Participants from Sydney underwent screening for sexually-transmitted infections: serological testing for syphilis and nucleic acid amplification testing of swabbed mucosae for chlamydia, gonorrhoea, and, where symptomatic, herpes simplex virus. Route of HCV acquisition was assigned by the clinician. If sexual and injecting risks coexisted within the twelve months preceding the study visit, the IDU route was assigned (‘IDU acquisition group’). If only sexual risk was present over this period, the sexual route was assigned (‘sexual acquisition group’). All data were de-identified. The study was conducted according to the Helsinki Declaration and approved by the Research Ethics Committee of St Vincent’s Hospital, Sydney.

### HCV sequencing

A 10mL EDTA blood sample was separated into plasma and cells. HCV RNA was quantified using an in-house PCR as described elsewhere [20]. Sequencing was attempted on samples with detectable HCV RNA, as determined by a qualitative HCV RNA assay (TMA assay; Versant, Bayer; lower limit of detection 10 IU/mL). Complementary DNA was generated using SuperScript VILO™ cDNA Synthesis Kit (Life Technologies, Carlsbad, CA) with random hexamers. A fragment of the HCV genome covering Core, Envelope-1, Hypervariable Region-1 (HVR1) and the beginning of Envelope-2 (E2) was amplified using a method previously described [21]. A fragment covering NS5B was also amplified, using a previously-described method, with modifications shown in S4 File [22]. Purified amplicons for both regions were sequenced using the Sanger method and sequence chromatograms processed using RECall: an automated sequence analysis pipeline [23]. Subtypes were determined using a HCV Automated Subtype Tool [24].

### Phylogenetics

As non-homologous sequences with sequence similarity owing to convergent evolutionary processes (homoplasies) can cause incorrect clustering when using short genomic fragments, HVR1 was removed from Core-E2. Sequences were aligned in Bioedit [25]. NS5B fragments were aligned and concatenated to Core-E2 to increase total sequence length and phylogenetic signal. Among participants with HCV genotypes (GT) 1a, 2b, 3a, and 4a, phylogenetic trees of the concatenated sequences were inferred using a maximum-likelihood approach implemented in RAxML through the CIPRES Gateway under the General Time Reversible model of nucleotide substitution with rate heterogeneity and the gamma model with invariant sites [26]. In order to support identification of ‘local’ clusters, 131 Australian and 93 Canadian sequences from HIV-positive or HIV-negative individuals with AHCV or chronic HCV (CHCV) infection were included as reference strains. Additional reference sequences were obtained from the Los Alamos National Laboratory database [27, 28] and aligned to the study sequences in Clustal X [29]. The robustness of the resulting tree was assessed using a rapid bootstrap algorithm with 1000 replicates. Clusters were identified using ClusterPicker with a bootstrap threshold of 90% and a maximum genetic distance threshold (GDT) of 0.05. These initial parameters were the same as those described elsewhere [30]. A sensitivity analysis was performed by varying the GDT to 0.03, 0.04, 0.06 and 0.07. Cut-offs were used to determine phylogenetic clusters before assessing the effect on the relationship with the network.

Time-scaled phylogenies for the same concatenated sequences were inferred using the Bayesian MCMC method implemented in BEAST v1.8.0 [31]. The substitution rates for Core-E2 and NS5B were estimated using a genomic partition model from an independent set of HCV isolates exhibiting strong temporal signal [32]. These rate estimates were subsequently applied as strong prior distributions on the molecular clock rates for the two genomic regions. Additionally, a codon-structured nucleotide substitution model [33], a relaxed clock with a log-normal distribution [34] and a Bayesian skyline coalescent prior [35] were employed. Clusters were determined from the time-scaled phylogenies if they had a posterior support of > 0.95 [31]. These clusters were compared to those identified in the RAxML trees and the time to most recent common ancestor (TMRCA) for each cluster was estimated using the time-scaled phylogenies.

### Venue-based network analysis

Venues used for sourcing sexual partners in the preceding six months were identified from interviews. Venues included physical venues including sex on premises venues (SOPV), websites and mobile phone apps. Sourcing partners from the same physical venue was considered a potential social link; sourcing partners from the same website was considered a potential link if participants were in the same city. While individuals may have sourced partners through specific physical venues, sexual contact could have occurred elsewhere (for example, a private home). Two-mode networks were constructed in UCINET 6.341 to illustrate links between individuals and venues [36]. These were transformed into one-mode networks to illustrate links between individuals sourcing partners at the same venues.

### Statistical analyses

Characteristics of individuals who were members of a pair/cluster were compared to those of non-clustering participants using Chi-squared, Fisher’s exact and Kruskal-Wallis tests. A Jaccard similarity coefficient (JSC), a measure of the similarity between two binary variables, was used to measure the association between the venue-based network and transmission relationships defined by phylogenetically-determined clusters (i.e. ≥ 2 participants with HCV genome sequence satisfying bootstrap and GDT requirements). The statistical significance of observed JSCs was assessed using the quadratic assignment procedure (QAP) with 10000 permutations. QAP is a method appropriate for assessing the statistical associations in relational data, where observations are on pairs of participants rather than individuals, and are therefore not independent. QAP generates data that correspond to the null hypothesis–that is, data that include no association between the HCV phylogeny and the venue-based network—by permuting the rows and columns using a random seed [37].

## Results

Clinical and demographic data for all forty participants are shown in Table 1, overall and separately by HCV acquisition risk. Median age was 45 years (IQR 35–50). The median duration of HCV infection at screening was 13 weeks (IQR 8–22).

**Table 1:** Comparison of characteristics for HIV-positive GBM with IDU versus sexually acquired acute HCV as determined by the clinician.

n = 40	IDU acquisition	Sexual acquisition	All
**Total number of participants**	15 (37.5)	25 (62.5)	40 (100)
**Median age, years**	37.6 (33.2–46.8)	46.0 (37.6–46.8)	45.3 (34.6–49.8)
**Australian born**	13 (86.7)	19 (76.0)	32 (80.0)
**City of recruitment**			
Sydney	11 (73.3)	15 (60.0)	26 (65.0)
**Estimated duration of HCV infection at screening, weeks**			
**median**	12.9 (7.7–24.4)	12.2 (7.1–22.4)	12.7 (7.8–21.3)
**<24**	11 (73.3)	20 (80.0)	31 (77.5)
**Any IDU, weeks**	15 (100.0)	5 (20.0)	20 (50.0)
>48 ago	0	5 (20.0)	5 (12.5)
>24 and ≤48 ago	1 (6.7)	0	1 (2.5)
> 4 and ≤24 ago	8 (53.3)	0	8 (20.0)
≤ 4 ago	6 (40.0)	0	6 (15.0)
**Injecting frequency**^**1**^			
Less than weekly	11 (78.6)	NA	x
**Use of a sterile needle and syringe for each IDU episode**^**2**^	5 (83.3)	NA	x
**Most frequently injected drug**^**1**^			
Methamphetamine	13 (86.7)	NA	x
**Permucosal drug use**^**1**^^**,**^^**3**^	10 (66.7)	14 (56.0)	24 (60.0)
**HCV seroconversion illness**			
Symptomatic	9 (60.0)	14 (56.0)	23 (57.5)
Jaundice	2 (13.3)	7 (28.0)	9 (22.5)
**Participant reported route of HCV acquisition**			
IDU	4 (26.7)	0	4 (10.0)
Sexual	11 (73.3)	25 (100)	36 (90.0)
**HCV RNA level, log IU/mL**	5.8 (3.3–6.2)	5.6 (4.7–6.2)	5.7 (4.2–6.2)
**Genotype 1a**	8 (53.3)	17 (68.0)	25 (62.5)
**Genotype 3a**	5 (33.3)	6 (24.0)	11 (27.5)
**Other or missing genotype**^**4**^	2 (13.3)	2 (8.0)	4 (10.0)
**Spontaneous clearance**	1 (6.7)	2 (8.0)	3 (7.5)
**HIV parameters**			
CD4 cell count, cells/mm^3^	530.0 (319.0–768.0)	527.0 (387.0–700.0)	528.5 (351.8–701.0)
Number receiving cART	9 (60.0)	20 (80.0)	29 (72.5)
No. on cART with HIV RNA <50 IU/mL	7 (77.8)	17 (85.0)	24 (82.8)
HBsAg positivity	1 (6.7)	0	1 (2.5)
**Median ALT, U/L**	81.0 (48.0–417.0)	149.0 (78.5–466.5)	134.5 (68.0–451.5)
**STI screen performed at baseline**	8 (53.3)	10 (40.0)	18 (45.0)
STI identified	4 (50.0)	4 (40.0)	8 (44.4)
**Number providing a detailed sexual history**^**1**^	12 (80.0)	22 (88.0)	34 (85.0)
Median number of sexual partners	22 (9–33)	9 (4–39)	15 (4–38)
**Number of participants reporting:**			
Sex exclusively with HIV+ partners	4 (33.3)	4 (18.2)	8 (23.5)
Group sex	10 (83.3)	14 (63.6)	24 (70.6)
Fisting	7 (58.3)	7 (31.8)	14 (41.2)
CRAI	10 (83.3)	17 (77.3)	27 (79.4)
CIAI	9 (75.0)	13 (59.1)	22 (64.7)
Injecting drugs around the time of sex	9 (75.0)	0	9 (26.5)

1, 2 With reference to the six months and one month preceding the study visit, respectively.

3 Drugs were ecstasy, MDMA, methamphetamine, GHB, cocaine, LSD, speed, magic mushrooms or marijuana either ingested, inhaled, or applied per rectum

4 One patient each of genotypes 1b, 2b and 4 (subtype not known); one patient not able to be genotyped due to low HCV RNA level

CRAI or CIAI denote condomless receptive or insertive anal intercourse, respectively

Brackets denote % or IQR.

Median HCV RNA level was similar for both groups (5.8 and 5.6 log IU/mL for IDU and sexual groups, respectively). GT1a (62.5%) and GT3a (27.5%) were most prevalent. Most individuals (57.5%) had symptomatic HCV seroconversions although jaundice was infrequent (22.5%). Three (7.5%) spontaneously cleared HCV. The median CD4 cell count was 529 cells/mm^3^; 72.5% took antiretrovirals, of whom 82.8% were HIV aviraemic. Of the 26 Sydney participants, 18 (69.2%) were tested for STIs, of whom 8 (44.4%) had an STI diagnosed. Eighty-five percent (n = 34) completed detailed interviews; 79.4% reported condomless receptive anal intercourse (C(R)AI), 60.0% group sex, 41.2% fisting, and 26.5% IDU during sex; 23.5% exclusively practised serosorting with other HIV-positive GBM.

Fourteen (35.0%) participants reported current (≤ six months prior to the study visit) IDU, whilst 20 (50.0%) reported having injected drugs at least once; 5/6 (83.3%) who had injected within the previous month reported always using a new sterile needle and syringe. Clinicians considered sexual transmission likely in 25 (62.5%) and IDU transmission in 15 (37.5%) men. In the clinician-assigned sexual transmission group, five (5/25, 20.0%) reported a lifetime history of IDU; none reported current injecting. Despite admitting current injecting, 11 (73.3%) men in the IDU group considered they had acquired HCV sexually. For the IDU group, methamphetamine was the most frequently injected drug (13/15, 86.7%). Fourteen (56.0%) in the sexual transmission group and 10 (66.7%) in the IDU group reported current permucosal drug use; of those specifying drug types, methamphetamine was most used (6/14, 42.9%). Most in the IDU group reported injecting less than weekly (11/14, 78.6%), and most reported injecting during sex. The median number of sexual partners in the preceding six months was greater for the IDU versus the sexual acquisition group (22 versus 9, respectively). Detailed IDU behaviours were available for 10/11 (90.9%) men for whom there was a discrepancy between clinician and patient assigned mode of HCV acquisition (S1 Table): over the preceding six months, the median number of injecting episodes was six (IQR 4–21) compared to 27 (10–33) sexual partners.

### Phylogenetics

**Fig 1:**
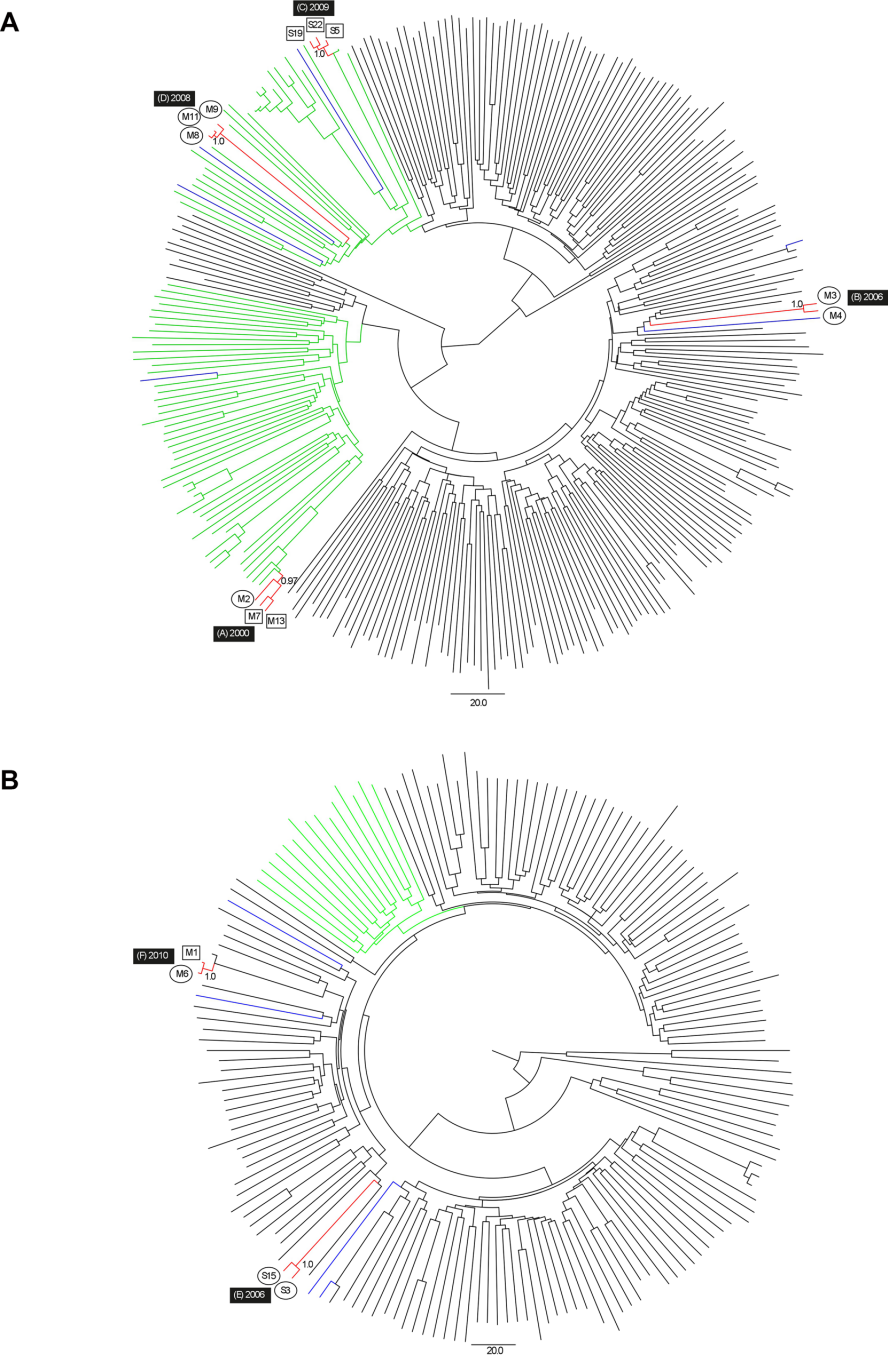
**Bayesian time-scaled phylogenies for HCV genotypes 1a and 3a (upper and lower panels respectively).** Red and blue denote sequences from this study that were clustered and non-clustered, respectively. Green denotes other Australian sequences within broader transmission lineages. Participants from Sydney and Melbourne are denoted by ‘S’ and ‘M’ respectively. Circles and squares denote sexual and IDU routes of HCV acquisition, respectively. Clusters are denoted by letters A to F, together with the corresponding time to most recent common ancestor (TMRCA). The scale bar represents time in years.

HCV sequencing was available for 28/40 (70.0%) participants, with low HCV RNA (<3 log IU/mL) or technical difficulties accounting for sequencing failures for seven and five participants respectively. Fifteen (15/28, 53.6%) sequences were grouped within three pairs and three clusters of three individuals (Fig 1). Four pairs/clusters comprised individuals sharing the same mode of HCV acquisition, whilst two contained individuals with a mixture of IDU and sexual modes. Individuals within the same pair/cluster were invariably recruited from the same city. The most recent common ancestor of all pairs/clusters was dated to 2000 or later.

Factors associated with being in a pair/cluster are shown in Table 2. Recruitment from Melbourne was significantly associated with clustering (p = 0.002). Age, HCV acquisition route, number of sex partners, IDU, non-injected drug use, CRAI, group sex, fisting, presence of STI, HCV genotype and HIV aviraemia were not associated with clustering.

**Table 2:** Factors associated with membership of a phylogenetic pair or cluster.

	Not in a pair or cluster	Within a pair or cluster	p value
**Total no. of individuals**	13 (47.4)	15 (53.6)	-
**Age, years**	45.3 (35.8–49.3)	44.8 (35.0–50.5)	0.882
**Site of recruitment**			
Melbourne	1 (6.67)	10 (76.9)	0.002^1^
**Recreational drug use**			
Injection drug	8 (61.5)	6 (40.0)	0.450
Non-injection drug	6 (46.2)	8 (53.3)	0.705
**Sexual History**^**2**^			
Median no. of sexual partners	23 (8–44)	7 (4–25)	0.122
Condomless receptive anal sex	8 (72.3)	11 (78.6)	1.000
Group sex	7 (63.6)	10 (71.4)	1.000
Fisting	4 (36.4)	8 (57.1)	0.302
**STI**^**3**^	4 (40.0)	0	0.497
**Route of HCV acquisition**			
Sexual	8 (61.5)	9 (60.0)	1.000
**HCV genotype**			
1a	6 (46.2)	11 (73.3)	0.246
**HIV RNA level <50 IU/mL**	8 (61.5)	7 (46.7)	0.476

1 p<0.05

2 For 25 individuals reporting detailed sexual behaviours within the six months prior to the study visit

3 For 13 individuals undergoing STI testing

Brackets denote % or IQR. Statistical analysis was performed with Mann-Whitney U, Chi-squared or Fishers exact tests.

### Venue-based network

**Fig 2:**
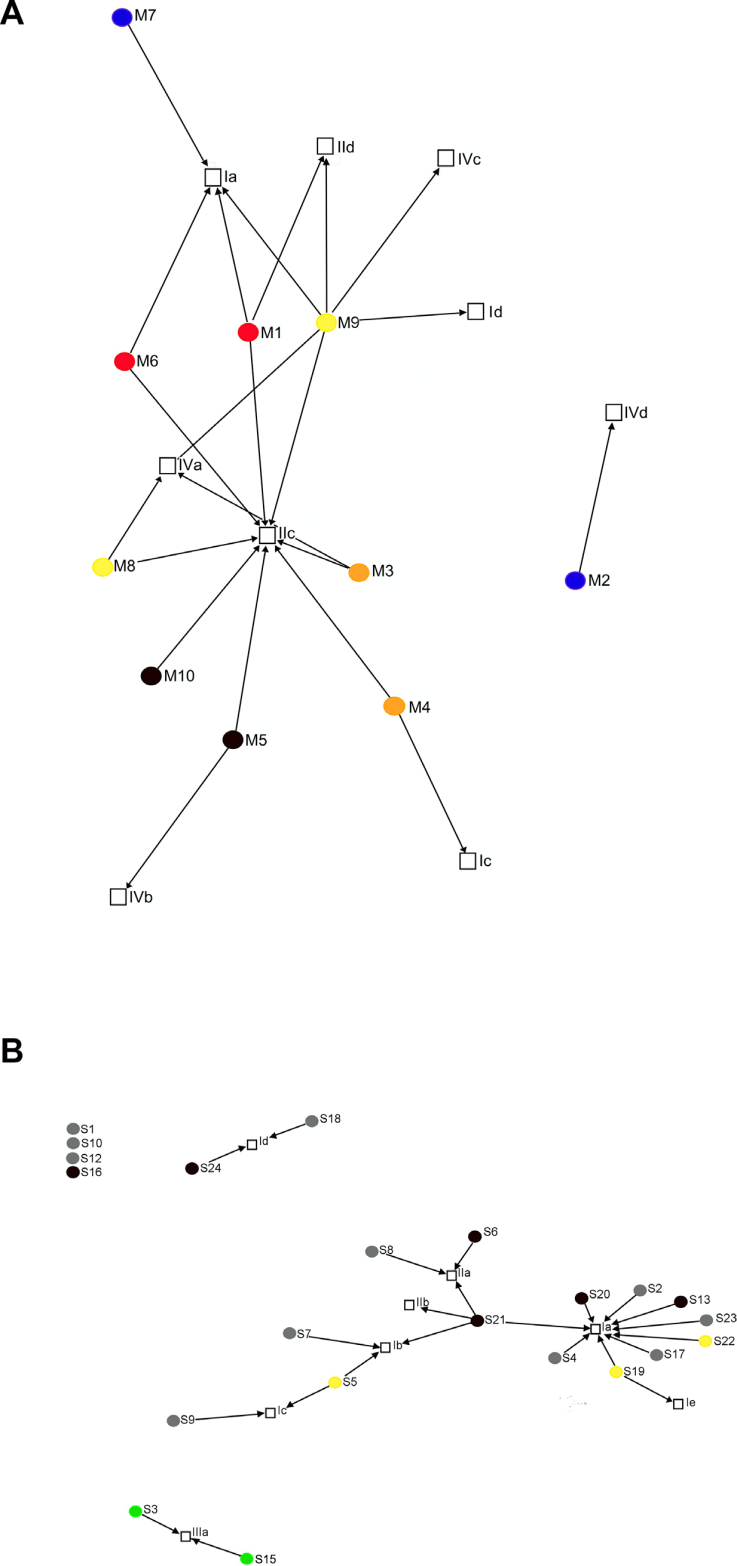
**Two-mode network diagrams for Melbourne and Sydney (upper and lower figures respectively).** Square boxes denote ‘venues’ used for sourcing sexual partners (I, websites or mobile phone aps; II, sex on premises venues; III, mutual friends; IV, bars or clubs). Circles denote participants from Sydney (S) or Melbourne (M). Colours denote membership of a phylogenetic pair or cluster; grey indicates that the participant is not in a cluster; black indicates that the participant is not included in the phylogeny. Arrows indicate links between participant and venues; participants who provided only generic information about venues used (rather than naming a specific venue) are shown as unlinked.

Data regarding venues used for sourcing sexual partners were available for 32/40 (80.0%) individuals; of these, ten were from Melbourne and twenty-two from Sydney. The remaining eight individuals (20.0%) declined to participate in the behavioural interview. The two-mode network diagrams are shown in Fig 2. Venues included SOPV and virtual venues. Most men (90.6%) nominated ≤2 venues. Using both physical and virtual venues was unusual, reported by only 15.6% of participants. In Melbourne, one SOPV was nominated by 8/10 participants. Five individuals in Melbourne nominated only physical venues, one only virtual venues and four nominated both venue types. In Sydney, fourteen (63.6%) individuals nominated only virtual venues (including a bareback website in eight cases), five (22.7%) nominated physical venues, one (4.5%) both physical venues and a bareback website, and two (9.1%) met via friends.

Table 3 shows the association between one-mode networks and phylogenetic clustering. There was no significant association for either the Sydney network or the Melbourne network and clustering (JSC 0.100, p = 0.17 and JSC 0.158, p = 0.57 respectively). Combining both networks, there was a strong association between the network and clustering (JSC 0.128, p = 0.005). The JSC was unchanged for the different genetic thresholds except for Melbourne when p≤0.03 (S2 Table).

**Table 3:** Association between 1-mode networks and phylogenetic clustering.

Participants	Jaccard similarity coefficient	Mean	SD	Min	Max	p-value
Melbourne	0.158	0.133	0.029	0.100	0.158	0.573
Sydney	0.100	0.045	0.045	0.000	0.222	0.168
All	0.128	0.033	0.025	0.000	0.158	0.005

## Discussion

This is the first molecular epidemiological study to investigate the potential influence of venue-based networks on HCV transmissions amongst HIV-positive GBM. Networks were defined by sourcing sexual partners through the same venues. Many men linked within the network belonged to phylogenetically-determined clusters, suggesting that HCV transmissions occurred between them, or that infecting strains shared a recent common ancestor. The widespread use of a bareback internet site (as indicative of interest in CAI with casual partners) and SOPVs is consistent with HIV-positive GBM at risk for HCV being engaged in a more sexually adventurous subculture [15]. These findings are important as they will help direct the targeting of public health interventions to reduce transmissions.

The Melbourne and Sydney networks were non-overlapping, with the former centred on men who attended one SOPV. While individuals also sourced partners from other sites, mixing may have occurred via links made at this venue (although it was not explicitly targeted at HIV-infected GBM or those seeking CAI). The Sydney network was more dispersed, with a bareback website being nominated by 40.9% of men. This may reflect differences in sexual behaviour or bias introduced by the small sample size. Overall, most participants sourced partners from one venue type but 15.6% used both websites and SOPVs. This highlights the potential number of linked contacts and suggests that strategies to reduce HCV incidence might involve venue-based health promotion.

We observed a similar proportion of phylogenetic clustering in the current study to that observed in the Australian Trial in Acute Hepatitis C (ATAHC) (54% and 51% respectively) [38]. New clusters were identified up to eight years after those reported in ATAHC (TMRCA 2000–2010 versus 1996–2002), suggesting recent HCV transmissions in HIV-infected GBM in Australia. Clusters were invariably centred on one city, demonstrating that the epidemics are occurring in parallel in different locations, as previously described [39]. Two pairs/clusters contained a mixture of individuals with IDU and sexual modes of HCV acquisition, consistent with findings from ATAHC that social mixing of HIV-infected GBM involving both IDU and non-IDU is likely to be contributing to the epidemic [38].

More clustering was seen for participants from Melbourne than from Sydney (p = 0.002), perhaps reflecting the finding that the Melbourne network was centralised around users of one SOPV. Attendance at this venue may have facilitated the development of new clusters, although we were unable to determine if high risk individuals preferred to attend at specific times. More likely, however, the results probably reflect increased numbers of recruits from the same network in Melbourne who were therefore members of the same clusters, whilst in Sydney many participants from the same network may have been missing.

Although there were associations between the phylogeny and network for both Sydney and Melbourne, these were not statistically significant, perhaps due to low numbers of participants. Combining the networks showed a significant association between the phylogeny and network (p = 0.005). This raises the possibility that venue-based social connections are important for HCV transmissions in individuals practising risky behaviours. For example, the venue-based network might constitute a sexual field–sexual preferences being influenced by participation in a particular venue, either due to peer norms or because some venues make particular sexual practices more feasible. In this presumably mutually reinforcing bidirectional relationship between sexual preferences and venues/subcultures, the sexual field could favour sexual practices associated with HCV transmissions [17].

Alternatively, the significant association between clustering and the combined network could reflect the observation that the two cities have non-overlapping networks, or the effect of geographic separation, as one physical venue cannot be in multiple locations. A larger study recruiting individuals common to both networks might allow these alternative possibilities to be distinguished, as well as the contribution of geographic versus social network distance. Of note, however, the sensitivity analysis demonstrated that any association between clustering and the network is unlikely to be dependent on the method used for defining clusters.

Two thirds (63%) of participants were determined by the clinician to have acquired HCV sexually. This rose to 90% if determined by the patient. In those individuals self-assigning a sexual HCV acquisition route for whom the clinician assigned an IDU route, four times as many sexual partners as episodes of IDU were reported. Participants always reported use of clean needles but multiple episodes of CRAI. This suggests that risky IDU may be relatively rare and risky sex more frequent. Of note, permucosal drug use was more common than IDU (60.0% versus 35.0% respectively) and may drive transmission through mucosal damage or behavioural disinhibition [7–9]. Individuals who reported current IDU reported over twice as many sexual partners as those without IDU, suggesting the former represent a very high ‘multiple risk’ group perhaps because of increased sexual disinhibition following IDU. This highlights the need to include health promotion around both safer injecting and sexual practices.

The observation that 24% of participants reported exclusive HIV serosorting is noteworthy given the current absence of data on the role of serosorting in incident HCV. As the prevalence of HCV is considerably higher amongst HIV-infected than–uninfected GBM in Australia (9.4% versus 1.1% respectively), serosorting among HIV-infected men is likely to increase risk of HCV transmission [40]. Previous studies have shown increasing levels of serosorting amongst GBM, possibly driven by the widespread availability of cART [41–43]. Many websites for meeting partners provide the means to specify partners’ HIV-serostatus and preferences for sex with or without condoms. Although we were unable to test for any link between serosorting and CAI, bareback website-users would seem important as a focus for providing information on risk reduction. This would also be consistent with findings from a survey of 947 GBM in Toronto: men who reported CAI and who considered themselves to be part of a ‘bareback scene’, as opposed to those who reported CAI unrelated to barebacking, were more likely to attend particular venues as well as report a distinctive set of beliefs around barebacking based on a notion of the ‘rational, informed, consenting, responsible masculine actor’ [44].

Stabilising HCV incidence rates in the Amsterdam HIV cohort may be due to improved HCV testing, increased HCV awareness or a saturation effect [45]. Public health approaches which apply these tools to venue-based networks could be effective, for example, links to relevant information on specific websites.

This study has limitations. First, there were relatively few participants, with potential recruitment bias. Individuals potentially belonging to clusters and the network may have been missed. The small sample size also limits the ability to detect statistically significant associations. Second, sequencing data were unavailable for 30% of individuals although this rate was comparable to a previous study using the same methodology and reflects the difficulty of generating Core-E2 amplicons through a nested approach [21]. Third, network data were based on venues where participants sourced sexual partners. Therefore potential IDU-related social links would have been missed. Network elicitation was conducted by asking about venues where partners were sourced rather than eliciting names of partners so the resulting inferred links represent probable rather than certain contacts. Fourth, men sourcing partners through particular venues may engage in riskier behaviour in private homes [46]. Nonetheless, health promotion could still be targeted through venue-based approaches. Finally, route of HCV acquisition as determined by the clinician was based on an assumption of an accurately self-reported sexual and IDU history; misclassification could have occurred, for example, in the context of undisclosed IDU. Similarly, ‘venues’ used for sourcing sex partners (eg SOPV, or meeting through a mutual friend) could not be independently verified. However, this problem is inherent to all behavioural studies where difficulties arise in trying to obtain collateral histories of sexual or drug-taking behaviour.

## Conclusions

This is the first study to investigate the relationship between the venue-based network and the phylogeny for HIV-infected GBM with recently-acquired HCV. The venues used were consistent with membership of a sexually adventurous gay subculture. The possibility is raised of an association between the network of sites where men source sexual partners and phylogenetic clustering, although a more important effect of geographical separation on clustering could not be excluded. Preventative messages targeted at the network may reduce transmissions, and these would need to be different according to local epidemiology. Intervention studies should assess the potential benefits of focusing public health strategies through network approaches.

## Supporting Information

S1 File Potential mechanisms for entry into study through (A) clinical hepatitis definition or (B) antibody seroconversion definition.
(PDF)

S2 File Study questionnaire instrument. (PDF)

S3 File Study interview schedule. (PDF)

S4 File Generation of NS5B amplicons. (PDF)

S1 Table Histories of injection drug use and sexual behaviour for ten men with discordance between clinician and participant-assigned mode of HCV acquisition. (PDF)

S2 Table Association between 1-mode social networks and phylogenetic clustering according to different genetic distance thresholds. (PDF)
