# Supplementary material for: Venue-Based Networks May Underpin HCV Transmissions amongst HIV-Infected Gay and Bisexual Men
Source: PLoS One. 2016 Sep 1;11(9):e0162002. doi: 10.1371/journal.pone.0162002 (PMC5008823; doi:10.1371/journal.pone.0162002)

Supplementary Information S1. Potential mechanisms for entry into study through (A) clinical hepatitis definition or (B) antibody seroconversion definition

Where X represents examples of clinical hepatitis in scenario A and antibody negative/antibody positive in scenario B

A

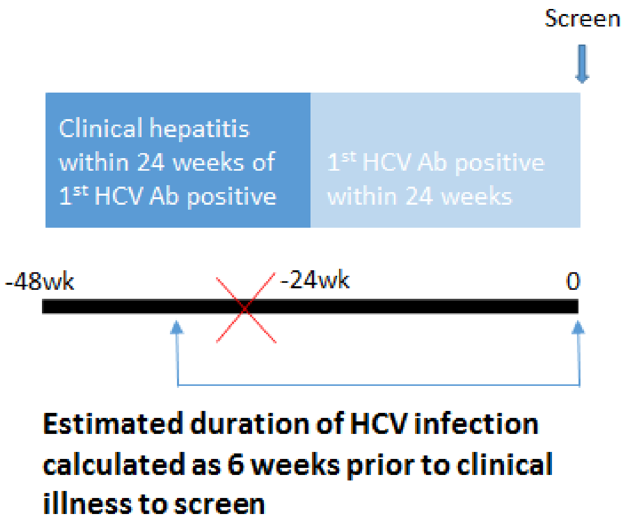

B

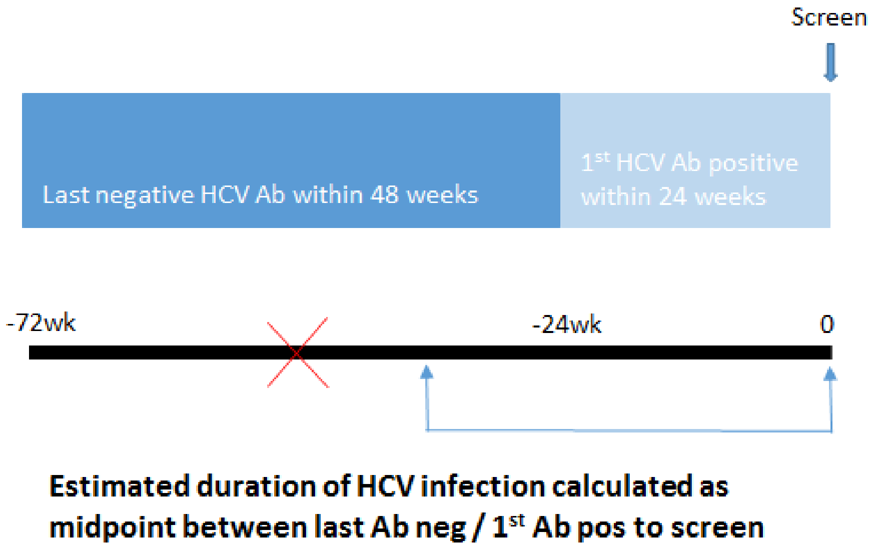

Supplement: S1 File — Potential mechanisms for entry into study through (A) clinical hepatitis definition or (B) antibody seroconversion definition. (PDF) [file pone.0162002.s001.pdf]
