# Supplementary material for: Venue-Based Networks May Underpin HCV Transmissions amongst HIV-Infected Gay and Bisexual Men
Source: PLoS One. 2016 Sep 1;11(9):e0162002. doi: 10.1371/journal.pone.0162002 (PMC5008823; doi:10.1371/journal.pone.0162002)
Supplement: S2 File — (PDF) [file pone.0162002.s002.pdf]

RAMPT-C

NATIONAL CENTRE IN HIV EPIDEMIOLOGY AND CLINICAL RESEARCH

Defining risk and mechanisms of permucosal transmission for acute HCV infection within high-risk populations

COMPLETION INSTRUCTIONS

- Ensure the patient information sheet and consent form has been correctly completed
- Ensure the visit date, study identifier are completed on each page of this form
- Complete all questions (unless otherwise specified)
- Except where specified, all questions are single response only
- Please write legibly
- All text and explanatory comments should be brief
- If the answer is zero, do not leave the field blank write '0'
- If the answer to the question is unknown, write 'not known' or 'NK'
- If a question is not applicable, write 'not applicable' or 'NA'
- Date format is dd/mm/yyyy
- If a date is partially known, enter that part of the date known and 'NK' for that not known part e.g. NK / 04 / 08
- When making a correction, draw a single line through the text to be changed so that the original entry is still legible. Initial and date the correction
- DO NOT use correction fluid or write over information as this will invalidate the data and create query

Study Schema

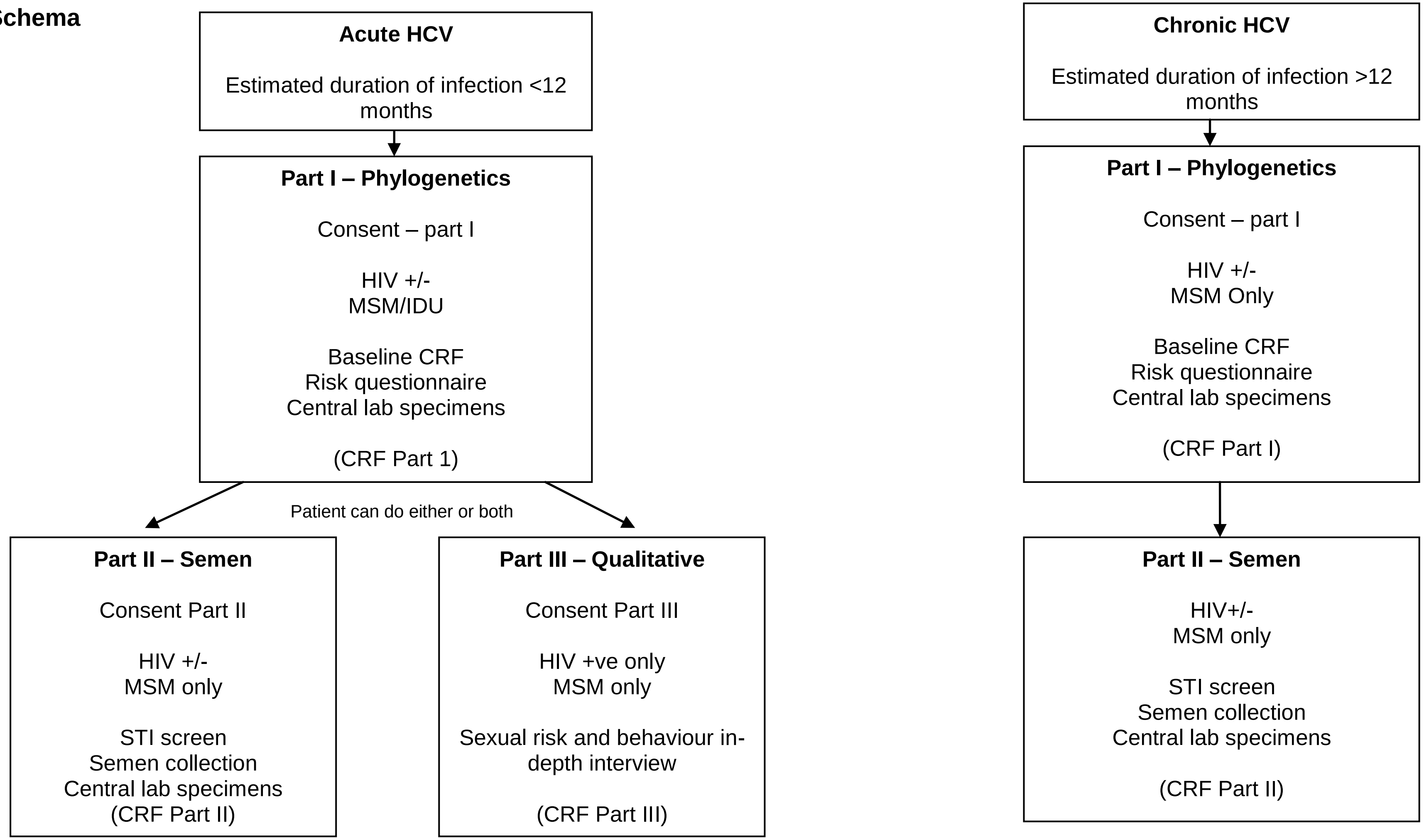

Schedule of Asessment – Baseline Visit

Schedule of Assessment	Part I only	Part I & II	Part I & III	Part I, II & III
Written Informed Consent	X	X	X	X
Central Lab – AMR<br>Stored Study Plasma Sample	X	X	X	X
Local Lab – standard of care				
• Anti-HCV antibody	X	X	X	X
• HCV RNA quantitative	X	X	X	X
• HCV genotype				
• BBV serology (HBsAG, HIV Ab)	X	X	X	X
• HIV RNA & T-cell subsets[a]	X	X	X	X
• SOC – LFT, platelets	X	X	X	X
Baseline Interview (includes Incident case interview & Sexual behavior and practices interview[b])	X	X	X	X
Central Lab – AMR<br>Stored Study Plasma Sample<br>Stored Semen Sample		 X<br>X		 X<br>X
Local Lab – STI Screen				
• Syphilis serology - SST tube		X		X
• Chlamydia, Gonorrhea, LGV - Urine, throat & rectal swabs		X		X
• HSV swab[c]		X		X
• HAV serology - SST tube		X		X
Behavioural Mechanisms Interview			X	X

[a] If HIV serology positive [b] Complete by acute MSM patients only [c] Perform in the presence of ulcerative lesions only

INCLUSION & EXCLUSION CRITERIA CHECKLISTS

Part I – Phylogenetics Component – ALL PATIENTS

All patients

Inclusion Criteria:

a) Is the male patient >= 16 years of age?

Yes □

No □

b) Has the patient provided written informed consent?

Yes □

No □

c) Is the patient HCV RNA positive?

Yes □

No □

d) Does the patient have a first positive HCV antibody or HCV RNA positive within the previous 6 months and documented HCV antibody negative or HCV RNA negative within the 12 months?

OR

Does the patient have a first positive HCV antibody or HCV RNA positive within the previous 6 months and documented acute clinical hepatitis (jaundice or ALT> 10x ULN)

Yes □

No □

e) Does the patient have adequate English to provide written, informed consent and to provide reliable responses to the study interview?

Yes □

No □

If any no box have been ticked the patient is **not eligible** to enter Part I of the Study

Exclusion criteria

a) Does the investigator consider the patient to be unlikely to participate in follow-up and/or unwilling to provide extra blood samples?

Yes □

No □

If the yes box have been ticked the patient is **not eligible** to enter Part I of the Study

PART II – Semen Component

N/A □ (please tick the box if patient is not enrolled into this Component)

Inclusion Criteria:
a) Is the male patient >= 16 years of age?
Yes □
No □
b) Has the patient provided written informed consent?
Yes □
No □
c) Is the patient HCV RNA positive?
Yes □
No □
d) Does the patient have a first positive HCV antibody or HCV RNA positive within the previous 6 months and documented HCV antibody negative or HCV RNA negative within the 12 months?
OR
Does the patient have a first positive HCV antibody or HCV RNA positive within the previous 6 months and documented acute clinical hepatitis (jaundice or ALT> 10x ULN)
OR
Has the patient been HCV RNA positive > 12 months (Chronic HCV)?
Yes □
No □
e) Does the patient have adequate English to provide written, informed consent and to provide reliable responses to the study interview?
Yes □
No □

If any no box have been ticked the patient is **not eligible** to enter Part II of the Study

Exclusion criteria:
a) Does the investigator consider the patient to be unlikely to participate in follow-up and/or unwilling to provide extra blood samples?
Yes □
No □
b) Is the patient currently on HCV treatment or had prior HCV treatment?
Yes □
No □

If any yes box have been ticked the patient is **not eligible** to enter Part II of the Study

PART III – Behavioural Interview Component

N/A □ (please tick the box if patient is not enrolled into this Component)

Inclusion Criteria:
a) Is the male patient >= 16 years of age?
Yes □
No □
b) Does the patient identify as MSM?
Yes □
No □
c) Is the patient co-infected with HIV?
Yes □
No □
d) Is the patient HCV RNA positive?
Yes □
No □
e) Does the patient have a first positive HCV antibody or HCV RNA positive within the previous 6 months and documented HCV antibody negative or HCV RNA negative within the 12 months?
OR
Does the patient has a first positive HCV antibody or HCV RNA positive within the previous 6 months and documented acute clinical hepatitis (jaundice or ALT> 10x ULN)
Yes □
No □
f) Does the patient have adequate English to provide written, informed consent and to provide reliable responses to the study interview?
Yes □
No □

If any no box have been ticked the patient is **not eligible** to enter Part III of the Study

Exclusion criteria
a) Does the investigator consider the patient to be unlikely to participate in follow-up and/or unwilling to provide extra blood samples?
Yes □
No □

If the yes box has been ticked the patient is **not eligible** to enter Part III of the Study

Interviewer's Name: ___________________________________

PART I – Phylogenetics Component (All patients)

Has subject been enrolled into the following cohort?	Yes	No
HITS-i	☐	☐
ATAHC	☐	☐
pH	☐	☐
HIM	☐	☐

HEPATITIS C DIAGNOSIS

1. Date first documented to be HCV antibody positive ___/___/______ dd/mm/yyyy

   ☐ not positive

2. Date first documented to be HCV RNA positive ___/___/______ dd/mm/yyyy

3. Date of most recent negative HCV antibody test ___/___/______ dd/mm/yyyy
   (Tick N/A ☐ if patient is chronic HCV)

   ☐ Never done
   ☐ No record of result

4. Documented HCV seroconversion illness?
   (Tick N/A ☐ if patient is chronic HCV)

   4a. If yes, which of the following symptoms have been experienced?

	Yes	No	Unclear/specify
Jaundice	☐	☐	☐ ____________________
Nausea/vomiting	☐	☐	☐ ____________________
Abdominal pain	☐	☐	☐ ____________________
Fever	☐	☐	☐ ____________________

   4b. Date of initial onset of symptoms ___/___/______ dd/mm/yyyy

5. Consent form signed	___/___/______<br>dd/mm/yyyy	
Components	Yes	No
Phylogenetics Study (Part I)	□	□
Semen Study (Part II)	□	□
Behavioural Interview (Part III)	□	□

VIROLOGICAL SCREENING

6. HCV Antibody — Sample collection date ___/___/______ (dd/mm/yyyy)

6a. HCV Antibody result ☐ Positive ☐ Negative ☐ Equivocal ☐ Not done

7. HCV RNA - Quantitative — Sample collection date ___/___/______ (dd/mm/yyyy)

7a. HCV RNA _______________ ☐copies/ml ☐ IU/ml

8. HCV Genotype — Sample collection date ___/___/______ (dd/mm/yyyy)

8a. Genotype _______________ Subtype ________________

9. HIV Antibody — Sample collection date ___/___/______ (dd/mm/yyyy)

9a. Anti-HIV result ☐ Positive ☐ Negative ☐ Equivocal ☐ Not done

10. HIV-1 RNA/CD4/CD8 — Sample collection date ___/___/______ (dd/mm/yyyy) ☐ Not required

10a. HIV RNA _______________ ☐copies/ml ☐ N/A

10b. CD4 total ___________ ☐cells/mm^3 CD8 total __________ ☐cells/mm^3 ☐ N/A

11. Hepatitis B Serology — Sample collection date ___/___/______ (dd/mm/yyyy)

11a. HBsAg ☐ Positive ☐ Negative ☐ Equivocal ☐ Not done

12. SOC Bloods

Sample collection date ___/___/______
dd/mm/yyyy

Liver Function Test	Result	Unit
Albumin		g/L
Total bilirubin		U/L
ALT		U/L
AST		U/L
Alkaline phosphatase		U/L
GGT		U/L

Full Blood Count	Result	Unit (please tick box)
Platelets		10^9/L

CENTRAL LABORATORY COLLECTION

13. Sample collection date ___/___/______
dd/mm/yyyy

Collected	Yes	No	Comments
Stored Plasma	☐	☐	____________________

DEMOGRAPHICS

I'd like to ask you a few questions about yourself

14. Do you consider yourself to be male, female or transgender?

Male 1
Female 2
Transgender 3
Not stated/inadequately described 9

15. Do you identify as?
Heterosexual/Straight 1
Bisexual 2
Gay/Lesbian 3

16. What is your year of birth?

□□□□
(yyyy)

17. Are you of Aboriginal or Torres Strait Islander origin?

No response 1
Non-indigenous 2
Aboriginal 3
Torres Strait Islander 4
Both Aboriginal and Torres Strait Islander 5
Indigenous non-specified 6

18. What is the main language you speak at home?

English.. 1
Other 2
(Specify ....)

19. What is your country of birth?

....

Please fax to NCHECR on 02 9385 0876 when study visit is complete.

20. If not Australian born, in what year did you first come to Australia?

    ....

21. Have you ever been in prison/ Juvenile justice centre?

    Yes 1
    No... 2

22. If yes, have you been in prison or juvenile justice centre within the last 2 years?

    Yes 1
    No... 2

DRUG USAGE AND INJECTING BEHAVIOURS

We now need to ask you some questions about your drug use – when you first used drugs and how often and how you use drugs and alcohol now. I'll also be asking you about practices such as sharing needles and syringes and other injecting equipment. One of the aims of this study is to examine how people become infected with hepatitis C. Remember all of this information is confidential. Your answers will not affect the treatment you receive.

23. Have you ever injected any drug?

    No.. 0 (If no, go to Question 27)
    Yes .... 1 (If yes, go to Question 24)

24. How old were you when you first injected any drug?

    ....years

25. When was the last time you injected?

    Within the last month 1
    Between 1 and 6 months ago 2
    Between 6 and 12 months ago .... 3 (go to question 27)
    Between 1-2 years ago.. 4 (go to question 27)
    More than 2 years ago... 5 (go to question 27)

26. If you injected in the last 6 months, how often did you inject?

More than three times most days... 1
2-3 times most days.. 2
Once a day 3
More than weekly, not daily (uses between 1-6 days per week)........... 4
Less than weekly 5
Have not injected in the last 6 months 6

27. What do you consider the most likely way and other possible ways in which you became infected with hepatitis C?

Most likely way (Circle one response only)		Other possible ways (circle all that apply)
1 ………………………	Injecting drug use	……………………1
2 ………………………	Transfusion of blood products	……………………2
3 ………………………	Occupational (needle stick or other exposure)	……………………3
4………………………	Sexual exposure to a known HCV positive person of the same sex	……………………4
5 ………………………	Sexual exposure to a known HCV positive person of the opposite sex	……………………5
6………………………	Sexual exposure to persons of unknown HCV status of the same sex	……………………6
7 ………………………	Sexual exposure to persons of unknown HCV status of the opposite sex	……………………7
8 ………………………	Body piercing	……………………8
9 ………………………	Tattoos	……………………9
10 …………………….	Use of other recreational drugs (snorting/inhaling)	…………………..10
11 …………………….	Other, specify	…………………..11
(……………………….)		(…..………………..)

28. Have you injected in the last month?

No.. 0 (If no, go to question 31)
Yes 1

29. How often did you use a NEW sterile needle and syringe last month?

All injections.. 1
Most of the time 2
Half of the time 3
Some of the time 4
Not in the last month... 5

30. How many times in the last month have you used a needle and/ or syringe after someone else had already used it?

None.. 0
One time.. 1
Two times 2
3-5 times.. 3
6-10 times.. 4
More than 10 times.. 5

31. What was the *last drug* you ever injected and the drug you injected *most often* in last 6 months?

**LAST DRUG**		**MOST OFTEN LAST 6 MONTHS**
1	Heroin	1
2	Methadone	2
3	Other opiates	3
4	Methamphetamine	4
5	Amphetamine	5
6	Cocaine	6
7	Cocaine +heroin	7
8	Benzodiazepines	8
9	Buprenorphine	9
10	Other (specify) (..)	10
	Never injected	99

32. In the last six months have you taken drugs in other ways other than injecting?

	Yes	No	Times
Nasally	☐	☐	(……..)
Other (specify)	☐	☐	(…………………..)

Please fax to NCHECR on 02 9385 0876 when study visit is complete.

TO BE COMPLETED BY ACUTE HCV MSM ONLY

SEXUAL BEHAVIOUR AND PRACTICES

These questions are about your sexual practices over the last six months. You will be asked about your sexual practices including information about number of partners, HIV status and specific practices that could contribute to sexual transmission of HCV. Remember all of this information is confidential. Your answers will not affect the treatment you receive.

33. In the past six months, how many different men have you had sex with?
    None.. 0
    One 1
    2-5 men 2
    6-10 men 3
    11-50 men 4
    More than 50 men.. 5

34. In the past six months, how many men of the men you had sex with were men whose HIV status you did not know?

    …………………………………………………………….men

35. In the past six months, how many of the men had sex with were men you knew were HIV positive?

    …………………………………………………………….men

36. In the past six months, how many of the men you had sex with, were men you knew were HIV negative?

    …………………………………………………………….men

37. In the past six months have you been involved in group sex (sex involving more than two individuals)? If so how many times?

    No.. 0
    Yes 1

    …………………………………………………………….times

38. In the last six months how many men have you been involved with the following sexual practice?

Sexual behaviour*	Number of men whose HIV status you did not know	Number of men you knew were **HIV positive**	Number of men you knew were HIV-negative
• Oral sex – you sucked his cock			
• Oral sex – he sucked your cock			
• Anal sex – he fucked you with a condom			
• Anal sex – you fucked him with a condom			
• Anal sex – he fucked you without a condom			
• Anal sex – you fucked him without a condom			
• Fisting – you fisted him while wearing a glove			
• Fisting – you fisted him without wearing a glove			
• Fisting – he fisted you while wearing a glove			
• Fisting – he fisted you without wearing a glove			
• S/M or bondage without blood			
• S/M or bondage with blood			
• Injected drugs before or during sex			

Thank you for participating and answering these questions.

Do you have any comments you would like to make?

End of Part I

PART II – Semen Component

CENTRAL LABORATORY COLLECTION

Sample collection date ___/___/______
dd/mm/yyyy

Collected	Yes	No	Comments
Stored Plasma	☐	☐	____________________

Collected	Yes	No	Comments
Stored Semen	☐	☐	____________________

LOCAL LABORATORY COLLECTION

STI SCREEN

Sample collection date ___/___/______
dd/mm/yyyy

Syphilis serology

	Reactive	Non-reactive	Not done	
Syphilis antibody				
Confirmatory tests if syphilis antibody reactive				
	Reactive	Non-reactive	Not done	N/A
TPPA				
FTA-ABS				
Non treponemal tests	Titre	Not done	N/A	
RPR				
VDRL				

Please fax to NCHECR on 02 9385 0876 when study visit is complete.

STI SCREEN continued……..

Chlamydia urine, throat & rectal swab

	Detected	Not detected	Not done
Chlamydia urine PCR			
Chlamydia throat swab PCR			
Chlamydia rectal swab PCR			

Lymphogranuloma venereum (LGV) rectal swab – only perform if Chlamydia PCR was detected

	Detected	Not detected	Not done	N/A
LGV rectal swab PCR				

Gonorrhoea urine, throat & rectal swab

	Detected	Not detected	Not done
Gonorrhoea urine PCR			
Gonorrhoea throat swab PCR			
Gonorrhoea throat swab Culture (if detected, indicate 1+, 2+, 3+ or 4+ by ticking the box)	☐ 1+<br>☐ 2+<br>☐ 3+<br>☐ 4+		
Gonorrhoea rectal swab PCR			
Gonorrhoea rectal swab Culture (if detected, indicate 1+, 2+, 3+ or 4+ by ticking the box)	☐ 1+<br>☐ 2+<br>☐ 3+<br>☐ 4+		

HSV swab – only perform in the presence of an ulcerative lesion

	Detected	Not detected	Not done	N/A
HSV-1 DNA PCR				
HSV-2 DNA PCR				

Please fax to NCHECR on 02 9385 0876 when study visit is complete.

STI SCREEN continued…….

HAV serology

	Detected	Not detected	Equivocal	Not done
HAV total antibody				

End of Part II

PART III – Behavioural Interview Component

Has the patient been referred to Dr Garrett Prestage for the Behavioural mechanisms interview?

Referral date ___/___/______
dd/mm/yyyy

End of Part III
