# Supplementary material for: Venue-Based Networks May Underpin HCV Transmissions amongst HIV-Infected Gay and Bisexual Men
Source: PLoS One. 2016 Sep 1;11(9):e0162002. doi: 10.1371/journal.pone.0162002 (PMC5008823; doi:10.1371/journal.pone.0162002)
Supplement: S3 File — (PDF) [file pone.0162002.s003.pdf]

D. RAMPT-C Qualitative Interview Portion

Simply discuss with me the topics I ask you about in your own words. Again, there are no right or wrong answers ... simply the answer that makes the most sense for you at the time.

1. How do you think you may have contracted Hep-C? Was there a specific person or situation that you think may have led to your Hep-C infection, and what was it about that person or situation that makes you think that was the reason?
2. In the six months prior to your diagnosis for HEP-C, were you aware of HEP-C? If you were aware of HEP-C, what did you think it was ***at the time***? How did you think ***at the time*** it might affect you if you became infected?
3. If you were aware of HEP-C, were you taking any precautions to prevent contracting HEP-C? If not, why not? If you were ... what were they, how did you employ them, and how effective did you think ***at the time*** that they would be in keeping you free of HEP-C?
4. Where do you meet your sex partners? How would you describe the type of men you like to have sex with? Do the men you usually have sex with tend to also have sex with each other or know each other?
5. What type of sex do you enjoy? Do you engage in group sex or attend sex parties? And if so, do those events ever include men you've played with before or you knew prior to that occasion?
6. Do you use drugs recreationally? What kinds, and how often? Do you use drugs as part of sex (for instance, P-n-P or 'party and play' situations)? What kinds, and how often?
7. Are you aware of any of your sex partners or other men in your sexual networks who have Hep-C? How do you know that?
8. Do you worry that you might pass on HEP-C to your sex partners? What, if anything, do you do to avoid transmitting HEP-C to others? Do you talk to your sex partners about your HEP-C status? Do you include anything on your online profiles about Hep-C?

Is there anything else you would like to add?
