# Supplementary material for: Venue-Based Networks May Underpin HCV Transmissions amongst HIV-Infected Gay and Bisexual Men
Source: PLoS One. 2016 Sep 1;11(9):e0162002. doi: 10.1371/journal.pone.0162002 (PMC5008823; doi:10.1371/journal.pone.0162002)
Supplement: S4 File — (PDF) [file pone.0162002.s004.pdf]

S4 Generation of NS5B amplicons

Forward (positions 8250-8275) and reverse primers (8616-8638) were used as described previously (Murphy et al 2007). Final PCR reactions contained one times concentration HI-FI buffer, 0.02 unit/µL of Velocity Taq Polymerase, 250 µM dNTP (Bioline), 200nM forward primer (Integrated DNA Technologies), 400nM reverse primer, one times concentration of Polymate (Bioline) and cDNA at 1/10 of the final reaction volume. PCRs were performed in a total volume of 50 µL. Thermocycling conditions are described in the table below. PCR products were stored at -20°C until required.

Thermocycling conditions for generation of NS5B amplicons

**1 cycle**	98°C 2 mins
**40 cycles**	98°C 30 secs<br>56°C 30 secs<br>72°C 1 min
**1 cycle**	72°C 10 mins
**Hold**	4°C
