# Supplementary material for: Venue-Based Networks May Underpin HCV Transmissions amongst HIV-Infected Gay and Bisexual Men
Source: PLoS One. 2016 Sep 1;11(9):e0162002. doi: 10.1371/journal.pone.0162002 (PMC5008823; doi:10.1371/journal.pone.0162002)
Supplement: S1 Table — (PDF) [file pone.0162002.s005.pdf]

Supplementary S1 Table. Histories of injection drug use and sexual behaviour for ten men with discordance between clinician and participant-assigned mode of HCV acquisition.

Study ID	Last time injected	Number of episodes of IDU in previous six months	New needle used in past month	Last ever injected drug	Most frequently injected drug	All sexual partners in ≤ 6 months	URAI, no. of partners in ≤ 6 months	UIAI, no. of partners in ≤ 6 months
M1	Within the last month	60	Always	Methamphetamine	Methamphetamine	30	30	30
M7	Between 1 and 6 months ago	8	NA	Methamphetamine	Methamphetamine	23	15	0
M11	Between 1 and 6 months ago	6	NA	Methamphetamine	Methamphetamine	30	5	5
S5	Between 1 and 6 months ago	4*	NA	Methamphetamine	Methamphetamine	34	15	15
S19	Between 1 and 6 months ago	1*	NA	Methamphetamine	Methamphetamine	10	11	10
S20	Within the last month	6	Always	Methamphetamine	Methamphetamine	10	10	10
S21	Within the last month	6	Always	Methamphetamine	Methamphetamine	NA	NA	NA
S23	Between 1 and 6 months ago	3*	NA	Buprenorphine	Buprenorphine	8	0	3
S25	Within the last month	6	Always	Methamphetamine	Methamphetamine	NA	NA	NA
S26	Within the last month	70	Always	Methamphetamine	Methamphetamine	85	85	0
**Median**	**x**	**6 (4-21)**	**x**	**x**	**x**	**27 (10-33)**	**13 (6-26)**	**8 (1-14)**

* lifetime use of IDU

NA denotes data not available.
