# Supplementary material for: Venue-Based Networks May Underpin HCV Transmissions amongst HIV-Infected Gay and Bisexual Men
Source: PLoS One. 2016 Sep 1;11(9):e0162002. doi: 10.1371/journal.pone.0162002 (PMC5008823; doi:10.1371/journal.pone.0162002)
Supplement: S2 Table — (PDF) [file pone.0162002.s006.pdf]

Supplementary S2 Table. Association between 1-mode social networks and phylogenetic clustering according to different genetic distance thresholds.

Participant group	Jaccard similarity coefficient	Mean	SD	Min	Max	p-value
Melbourne p>0.03	0.158	0.133	0.029	0.100	0.158	0.573
Melbourne p<=0.03	0.167	0.103	0.034	0.050	0.167	0.146
Sydney	0.100	0.045	0.045	0.000	0.222	0.168
All p>0.03	0.128	0.033	0.025	0.000	0.158	0.005
All p<=0.03	0.132	0.029	0.025	0.000	0.162	0.004
